# Supplementary material for: Exosome Trafficking Is a Key Regulator of Adipocyte Thermogenesis
Source: bioRxiv. 2025 Jul 31:2025.07.20.665768. Preprint. [Version 2] doi: 10.1101/2025.07.20.665768 (PMC12324204; doi:10.1101/2025.07.20.665768)
Supplement: 1 [file NIHPP2025.07.20.665768v2-supplement-1.pdf]

bioRxiv preprint doi: https://doi.org/10.1101/2025.07.20.665768; this version posted July 31, 2025. The copyright holder for this preprint (which was not certified by peer review) is the author/funder, who has granted bioRxiv a license to display the preprint in perpetuity. It is made available under aCC-BY 4.0 International license.

Supporting Information Appendix (SI)

Figures

A

miRNA	p-value	fold change
hsa-miR-28-5p	0.0001	-83121.09
hsa-miR-519d-5p	0.0007	-5499.29
hsa-miR-221-5p	0.0018	-320.43
hsa-miR-27b-3p	0.0022	3553.45
hsa-miR-99b-5p	0.0039	1243.66
hsa-miR-26b-3p	0.0042	89.77
hsa-miR-378d	0.0073	-259.04
hsa-miR-328-3p	0.0083	-36.92
hsa-miR-532-5p	0.0089	-65.23
hsa-miR-454-3p	0.0093	103.45
hsa-miR-27a-3p	0.0105	45.88
hsa-miR-126-3p	0.0123	72.28
hsa-miR-34a-3p	0.0161	-24.42
hsa-miR-378a-5p	0.0188	-28.04
hsa-miR-107	0.0193	-14.04
hsa-miR-130b-3p	0.0194	12.41
hsa-miR-133a-5p	0.0198	33.18
hsa-miR-155-5p	0.0208	-69.76
hsa-miR-133a-3p	0.0222	-11.01
hsa-miR-30c-1-3p	0.0234	9.41
hsa-miR-138-1-3p	0.0249	-21.97
hsa-miR-16-2-3p	0.0251	-18.21
hsa-miR-196a-2	0.0272	11.61
hsa-miR-320a	0.0272	-4.85
hsa-miR-130a-3p	0.0280	5.06

miRNA	p-value	fold change
hsa-miR-221-3p	0.0284	43.90
hsa-miR-18a-5p	0.0296	6.25
hsa-miR-32-5p	0.0351	5.60
hsa-miR-365a-2	0.0363	2.28
hsa-miR-34c-5p	0.0411	9.78
hsa-miR-203a-3p	0.0435	4.94
hsa-let-7b-5p	0.0441	-3.18
hsa-miR-92a-1	0.0464	5.02
hsa-miR-192-3p	0.0471	-1.23
hsa-miR-100-5P	0.0503	4.43
hsa-miR-29b-3P	0.0504	2.32
hsa-miR-30b-5p	0.0504	3.46
hsa-miR-146b-5P	0.0510	-1.17
hsa-miR-365a-1	0.0543	2.15
hsa-miR-193a-3p	0.0550	8.35
hsa-miR-423-5p	0.0553	-1.10
hsa-miR-484	0.0560	3.37
hsa-miR-126-5p	0.0577	-1.10
hsa-miR-92a-2	0.0579	1.95
hsa-miR-10b-5p	0.0581	-8.31
hsa-miR-196a-5p	0.0589	-4.43
hsa-miR-328-3p	0.0603	-7.67
hsa-miR-21-3p	0.0611	-2.77
hsa-miR-222-5p	0.0612	-7.06
hsa-RNP-u6	0.0627	-1.05

miRNA	p-value	fold change
hsa-miR-150-3p	0.0647	1.72
hsa-miR-132-3p	0.0688	3.93
hsa-miR-99b 3p	0.0735	23.65
hsa-miR-193b-5p	0.0747	2.61
hsa-miR-378a-3p	0.0759	-1.12
hsa-miR-222-3p	0.0759	1.26
hsa-miR-30b-5p	0.0762	1.76
hsa-miR-25-3p	0.0768	-3.24
hsa-miR-192-5p	0.0777	-1.12
hsa-miR-30b-3p	0.0787	1.52
hsa-miR-133a-5p	0.0843	1.90
hsa-miR-21-5p	0.0865	5.55
hsa-miR-324-3p	0.0875	-2.69
hsa-miR-19a-3p	N.S.	2.97
hsa-miR-193b-3p	N.S.	1.15
hsa-miR-34c-3p	N.S.	4.14
hsa-miR-181b-5p	N.S.	-1.15
hsa-miR-30c-5p	N.S.	1.07
hsa-miR-16-5p	N.S.	2.64
hsa-miR-26a-5p	N.S.	-10.08
hsa-miR-374b-3p	N.S.	-31.04
hsa-miR-26b-5p	N.S.	-11.68
hsa-miR-455	N.S.	-1.74
hsa-miR-378c	N.S.	-2.44
hsa-miR-204-5p	N.S.	2.06

miRNA	p-value	fold change
hsa-miR-140-5p	N.S.	-1.70
hsa-miR-20a-5p	N.S.	2.08
hsa-miR-17-5p	N.S.	-1.82
hsa-let-7g-5p	N.S.	-1.07
hsa-miR-519d-3p	N.S.	-12.84
hsa-miR-33b-5p	N.S.	-4.73
hsa-miR-15a-5p	N.S.	9.34
hsa-miR-34a 5p	N.S.	3.33
hsa-miR-16-1-3p	N.S.	-3.72
hsa-miR-27b-5p	N.S.	15.19
hsa-miR-125b-5p	N.S.	-18.94
hsa-miR-106a-5p	N.S.	4.38
hsa-miR-150-5p	N.S.	4.74
hsa-miR-27a-5p	N.S.	-1.70
hsa-miR-30c-2-3p	N.S.	27.71
hsa-miR-144-5p	N.S.	-24.96
hsa-miR-365a-3p	N.S.	-21.38
hsa-miR-19a-5p	N.S.	-1.70
hsa-miR-142-3p	N.S.	8.02
hsa-miR-374b-5p	N.S.	-1.70
hsa-miR-150-5p	N.S.	-1.70
hsa-miR-138-5p	N.S.	1.07
hsa-miR-33a-5p	N.S.	-9.52

B

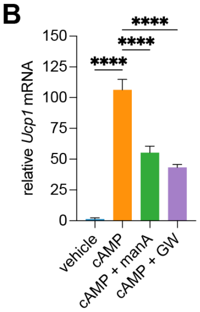

Fig. S1: (A) microRNA expression in exosomes from beige adipocytes under whitening versus forskolin-stimulated conditions. Human iPSC-derived beige adipocytes were differentiated for 12 days, then cultured under non-adipogenic (whitening) conditions for 4 days before treatment with forskolin for 6 hours. Exosomes were collected from culture media, and their microRNA content was quantified by targeted qPCR profiling of 98 microRNAs associated with adipogenesis, thermogenesis, and metabolic function. Differential expression analysis was performed using Global Pattern Recognition (GPR) software. This statistical algorithm makes no *a priori* assumptions about normalizers. Inspired by triangulation methods in cartography and astronomy, it iteratively compares each gene's expression relative to all others in a gene panel to establish a global expression pattern, identifying and ranking significant changes. The plot lists microRNAs with corresponding fold changes sorted by p-values, colored orange for upregulated and blue for downregulated microRNAs following forskolin stimulation compared to whitening conditions. Data represent the mean of $n = 3$ independent experiments. (B) Inhibition of exosome secretion with manumycin A or GW4869 attenuated cAMP-induced upregulation of *Ucp1* mRNA expression in beige adipocytes. Cells were pretreated with manumycin A (1 µM) or GW4869 (10 µM) for 2 hours prior to cAMP stimulation (500 nM) for 6 hours, and *Ucp1* transcript levels were measured by qPCR. These findings indicate that the observed effects are not due to off-target actions of forskolin.

bioRxiv preprint doi: https://doi.org/10.1101/2025.07.20.665768; this version posted July 31, 2025. The copyright holder for this preprint (which was not certified by peer review) is the author/funder, who has granted bioRxiv a license to display the preprint in perpetuity. It is made available under aCC-BY 4.0 International license.

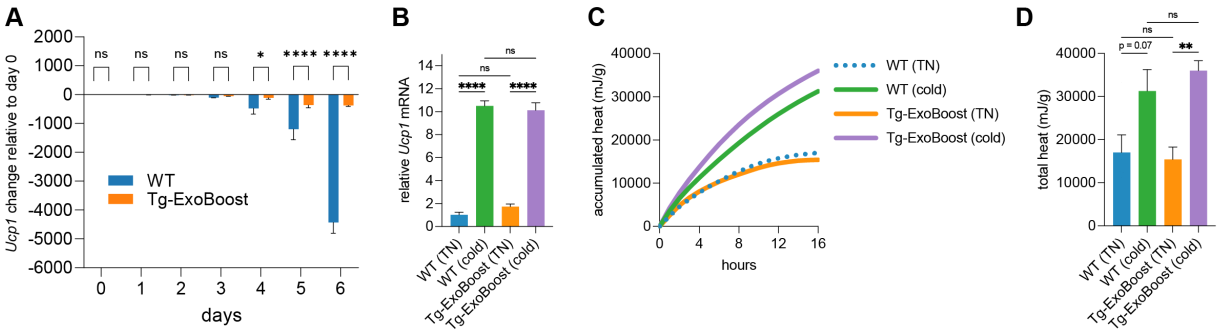

Fig. S2: (A) Tg-ExoBooster-positive beige adipocytes maintain *Ucp1* expression during whitening. *Ucp1* mRNA levels in differentiated wild-type (WT) and Tg-ExoBooster beige adipocytes (6 days) cultured in non-adipogenic basal medium for an additional 6 days. Tg-ExoBooster cells maintain higher *Ucp1* expression compared to wild-type. $n = 4$ biological replicates per group; unpaired t-test indicated. (B) Comparable thermogenic responses in brown adipose tissue of WT and Tg-ExoBooster female mice both fed doxycycline diet (3 days) followed by 3 days cold challenge (details in text). RT-qPCR analysis of *Ucp1* expression in brown adipose tissue from wild-type and Tg-ExoBooster mice after doxycycline exposure and cold challenge ($n = 5$). (C, D) Ex vivo microcalorimetry traces showing (C) average heat accumulation over time and (D) total heat output (quantified from C, with error bars) from inguinal fat pads post-cold challenge ($n = 5$). Heat data in C-D normalized to tissue weight in grams (g).

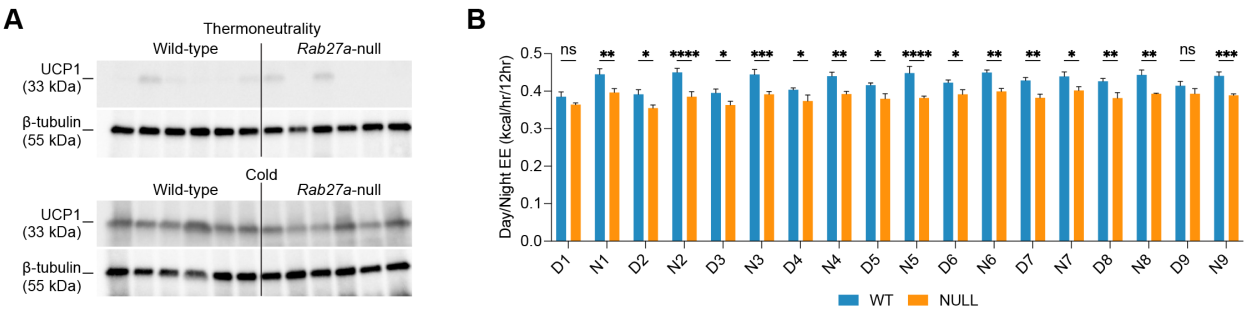

Fig. S3: (A) Western blot analysis of UCP1 protein expression in inguinal adipose tissue from wild-type and *Rab27a* null mice following 7 days at thermoneutrality (29 °C) and a subsequent 7-day cold exposure. Protein levels were normalized to -tubulin to control for loading. Data correspond to the experiments shown in Fig. 3H. (B) Day-by-day (D) and nighttime energy expenditure profiles (N) in wild-type and *Rab27a* null mice during the 9-day treatment period measured using metabolic cages. Corresponding average energy expenditure across the 9 days is shown in Fig. 4M.

bioRxiv preprint doi: https://doi.org/10.1101/2025.07.20.665768; this version posted July 31, 2025. The copyright holder for this preprint (which was not certified by peer review) is the author/funder, who has granted bioRxiv a license to display the preprint in perpetuity. It is made available under aCC-BY 4.0 International license.

Tables

Table S1: Metabolic and DXA analysis of WT vs *Rab27a* +/− mice (RT vs cold).

Parameter measured	Units	WT	Het	p-value
Body weight RT (day 7)	g	20.10	21.09	0.235
Body weight cold (day 7)	g	19.45	20.27	0.3589
Body weight difference (RT vs cold)	%	-3.22	-3.91	–
Fat mass RT (day 7)	g	2.53	3.20	0.0005
Fat mass cold (day 7)	g	2.35	2.48	0.4561
Fat mass difference (RT vs cold)	%	-6.93	-22.40	–
Lean mass RT	g	13.56	13.87	0.6407
Lean mass cold	g	13.12	13.65	0.4459
Lean mass difference	%	-3.29	-1.56	–
Daytime energy expenditure RT	kcal/14hr	0.34	0.39	0.6782
Daytime energy expenditure cold	kcal/14hr	0.82	0.76	0.0037
Nighttime energy expenditure RT	kcal/10hr	0.47	0.46	0.8856
Nighttime energy expenditure cold	kcal/10hr	0.93	0.88	0.0993
24h energy expenditure RT	kcal/24hr	0.43	0.44	0.5763
24h energy expenditure cold	kcal/24hr	0.88	0.82	0.0200
Daytime RER RT	%/14hr	0.87	0.86	0.3416
Daytime RER cold	%/14hr	0.91	0.91	0.8286
Nighttime RER RT	%/10hr	0.92	0.93	0.4217
Nighttime RER cold	%/10hr	0.94	0.95	0.1798
24h RER RT	%/24hr	0.90	0.89	0.9361
24h RER cold	%/24hr	0.92	0.93	0.4739
Daytime REE RT	kcal/30min/14hr	0.37	0.34	0.3357
Daytime REE cold	kcal/30min/14hr	0.73	0.68	0.0362
Nighttime REE RT	kcal/30min/10hr	0.37	0.34	0.3600
Nighttime REE cold	kcal/30min/10hr	0.79	0.73	0.0149
24h REE RT	kcal/30min/24hr	0.36	0.49	0.4919
24h REE cold	kcal/30min/24hr	0.76	0.70	0.0114
Daytime AEE RT	kcal/30min/14hr	0.49	0.49	0.8010
Nighttime AEE RT	kcal/30min/10hr	0.52	0.54	0.5392
24h AEE RT	kcal/30min/24hr	0.50	0.52	0.6286
Daytime AEE cold	kcal/30min/14hr	0.93	0.91	0.4956
Nighttime AEE cold	kcal/30min/10hr	1.02	0.95	0.1063
24h AEE cold	kcal/30min/24hr	0.97	0.93	0.1952
Food consumed/day RT	g/24hr	2.01	1.44	0.1801
Food consumed/day cold	g/24hr	3.60	3.07	0.2494
Food consumed/night RT	g/24hr	2.50	2.49	0.9746
Food consumed/night cold	g/24hr	3.32	3.36	0.9124
24h food consumed RT	g/24hr	4.51	3.92	0.2961
24h food consumed cold	g/24hr	6.92	6.43	0.4146
24h water consumed/day RT	g/24hr	3.63	3.16	0.2452
24h water consumed/day cold	g/24hr	3.62	3.49	0.7817
24h Y beam breaks (activity) RT	#/24hr	32314.10	31337.20	0.8103
24h Y beam breaks (activity) cold	#/24hr	33845.00	32542.00	0.7647
24h X beam breaks (activity) RT	#/24hr	22834.10	22445.00	0.8684
24h X beam breaks (activity) cold	#/24hr	23218.20	20482.70	0.2821
24h in cage walking meters RT	m/24hr	316.38	229.33	0.0462
24h in cage walking meters cold	m/24hr	236.00	197.67	0.3936
24h in cage walking speed RT	m/s/24hr	0.03	0.03	0.9505
24h in cage walking speed cold	m/s/24hr	0.03	0.03	0.3572
24h Hours of Sleep (¿40sec) RT	hrs/24hrs	11.88	12.33	0.7241
24h Hours of Sleep (¿40sec) cold	hrs/24hrs	11.93	11.10	0.5494

bioRxiv preprint doi: https://doi.org/10.1101/2025.07.20.665768; this version posted July 31, 2025. The copyright holder for this preprint (which was not certified by peer review) is the author/funder, who has granted bioRxiv a license to display the preprint in perpetuity. It is made available under aCC-BY 4.0 International license.

Table S2: Metabolic and DXA analysis of WT vs *Rab27a* −/− mice (CL316,243 treated).

Parameter measured	Units	WT	Null	p-value
Body weight RT (day 7)	g	19.05	18.78	0.6745
Body weight cold (day 7)	g	19.88	19.07	0.0323
Body weight difference (RT vs cold)	%	4.47	1.82	0.3770
Fat mass RT (day 7)	g	2.77	3.45	0.0270
Fat mass cold (day 7)	g	2.34	3.14	0.0347
Fat mass difference (RT vs cold)	%	-15.43	-12.21	0.5853
Lean mass pre CL (day 1)	g	12.56	12.16	0.4797
Lean mass post CL (day 9)	g	13.40	12.00	0.0087
Lean mass change	%	5.95	-0.10	0.0051
Daytime energy expenditure	kcal/14hr	0.43	0.39	0.0062
Nighttime energy expenditure	kcal/10hr	0.44	0.40	0.0014
24h energy expenditure	kcal/24hr	0.44	0.39	0.0020
Daytime respiratory exchange ratio	%/14hr	0.78	0.80	0.0056
Nighttime respiratory exchange ratio	%/10hr	0.89	0.89	0.6171
24h respiratory exchange ratio	%/24hr	0.83	0.85	0.1315
Daytime resting energy expenditure	kcal/30min/14hr	0.38	0.37	0.7722
Nighttime resting energy expenditure	kcal/30min/10hr	0.37	0.30	0.0326
24h resting energy expenditure	kcal/30min/24hr	0.38	0.34	0.0012
Daytime active energy expenditure	kcal/30min/14hr	0.49	0.48	0.6879
Nighttime active energy expenditure	kcal/30min/10hr	0.50	0.44	0.0003
24h active energy expenditure	kcal/30min/24hr	0.49	0.46	0.0980
Food consumed/day	g/24hr	4.63	4.01	0.2754
Water consumed/day	g/24hr	3.46	2.96	0.4984
24h Y beam breaks (activity)	#/24hr	23756.17	19361.50	0.1392
24h X beam breaks (activity)	#/24hr	23378.00	19429.17	0.1742
24h in cage walking meters	m/24hr	174.11	198.85	0.2781
24h in cage walking speed	m/s/24hr	0.03	0.03	0.2015
24h Hours of Sleep (¿40sec)	hrs/24hrs	0.03	0.03	0.2015
